# Online Extraction of Pose Information of 3D Zigzag-Line Welding Seams for Welding Seam Tracking

**DOI:** 10.3390/s21020375

**Published:** 2021-01-07

**Authors:** Bo Hong, Aiting Jia, Yuxiang Hong, Xiangwen Li, Jiapeng Gao, Yuanyuan Qu

**Affiliations:** 1 College of Mechanical Engineering, Xiangtan University, Xiangtan 411105, China; hongbo@xtu.edu.cn (B.H.); jat0929@163.com (A.J.); 201831540124@smail.xtu.edu.cn (J.G.); 201931000153@smail.xtu.edu.cn (Y.Q.); 2 College of Mechanical and Electrical Engineering, China Jiliang University, Hangzhou 310018, China; hongyuxiang@cjlu.edu.cn

**Keywords:** 3D zigzag-line welding seams, welding seam tracking, extraction of pose information of welding seams, laser displacement sensor, point cloud segmentation

## Abstract

Three-dimensional (3D) zigzag-line welding seams are found extensively in the manufacturing of marine engineering equipment, heavy lifting equipment, and logistics transportation equipment. Currently, due to the large amount of calculation and poor real-time performance of 3D welding seam detection algorithms, real-time tracking of 3D zigzag-line welding seams is still a challenge especially in high-speed welding. For the abovementioned problems, we proposed a method for the extraction of the pose information of 3D zigzag-line welding seams based on laser displacement sensing and density-based clustering point cloud segmentation during robotic welding. after thee point cloud data of the 3D zigzag-line welding seams was obtained online by the laser displacement sensor, it was segmented using theρ-Approximate DBSCAN (Density-Based Spatial Clustering of Applications with Noise) algorithm. In the experiment, high-speed welding was performed on typical low-carbon steel 3D zigzag-line welding seams using gas metal arc welding. The results showed that when the welding velocity was 1000 mm/min, the proposed method obtained a welding seam position detection error of less than 0.35 mm, a welding seam attitude estimation error of less than two degrees, and the running time of the main algorithm was within 120 ms. Thus, the online extraction of the pose information of 3D zigzag-line welding seams was achieved and the requirements of welding seam tracking were met.

## 1. Introduction

**Figure 1:**
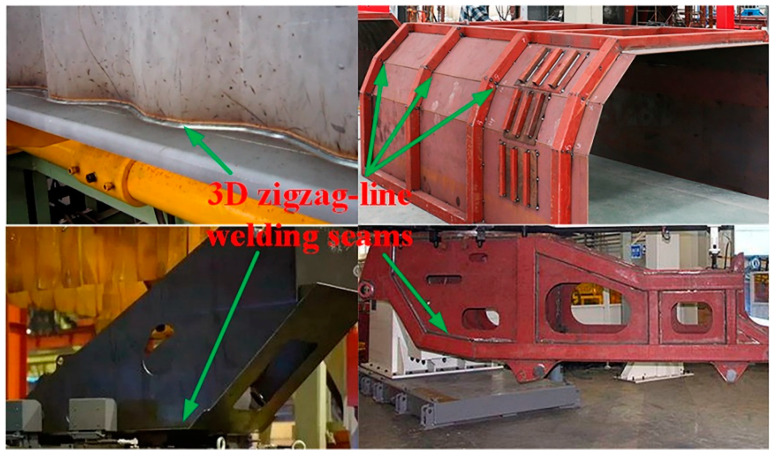
Examples of three-dimensional (3D) zigzag-line welding seams.

Three-dimensional (3D) zigzag-line welding seams are found extensively in the manufacturing of marine engineering equipment, heavy lifting equipment, and logistics transportation equipment (Figure 1). Workpieces with 3D zigzag-line welding seams are assembled by positioning the welding seams with zigzag lines for different fold angles, and the pose of the welding seam changes in real time during the welding process. The off-line robot programming mode and the robot teaching mode have their disadvantages, i.e., low programming efficiency and a heavy teaching workload, which seriously affect the welding efficiency and quality of 3D zigzag-line welding seams [1,2,3]. The real-time tracking of 3D zigzag-line welding seams is an effective way to improve the welding efficiency and quality of 3D zigzag-line welding seams. However, the welding seam tracking methods that are currently available are ineffective for tracking 3D zigzag-line welding seams in real time, especially in high-speed welding. The key to achieving real-time tracking of 3D zigzag-line welding seams is to develop a system for the online extraction of pose information for 3D zigzag-line welding seams.

Vision-based 3D automatic welding has become a popular research topic [4,5,6,7,8]. The methods most commonly used to extract the pose information for 3D welding seams are the binocular vision-based method, which is based on RGB-D (RGB-Depth) sensors, and a method based on laser structured light.

The binocular vision-based method perceives 3D information by simulating the function of the human eye, that is, depth information is sensed by parallax calculation [9]. Dinham et al. [10] proposed a method for autonomous welding seam identification and localization using eye-in-hand stereo vision for robotic arc welding, which detects 3D welding seams with an accuracy of 1 mm through the proposed identification method and a stereo matching algorithm. In a study by Jia et al. [11], a method for extracting depth information based on binocular stereo vision was proposed to calibrate a binocular camera, obtain a parallax map with the stereo matching method, and extract depth information based on the principle of trigonometric distance measurement.

The RGB-D sensor is a commonly used 3D measurement sensor and is widely applied to welding, mobile robots, and robot assembly. The RGB-D sensor can obtain an image of a welding environment as well as information about the depth of welding seams. Jing et al. [12] proposed an RGB-D sensor-based auto path generation method for an arc-welding robot. The pose information for the welding seams was extracted from the point cloud data of the welding workpieces. The weld path was generated by the auto path generation algorithm and used to assist the teaching of the welding robot, which improved the teaching efficiency of the welding robot. Silvers et al. [13] designed a human–machine interaction system based on the RGB-D visual sensor, which located the welding seams by identifying a human hand pointing to the welding seams, and the experimental results showed that this method effectively identified the welding seams. Using the RGB-D sensor to obtain the point cloud data, Peng et al. [14] proposed an automatic groove detection method based on the geometric characteristics of cloud point data and they generated 3D welding trajectories that were applicable to different V-groove welding workpieces.

Laser structured light sensors have been widely used in the 3D measurement of welding robots due to their high precision and great robustness. Zeng et al. [15] proposed a 3D path teaching method based on laser structured light. This method was used to apply laser fringe processing technology to extract 3D welding seams and to obtain the pose information of welding seams according to their 3D information. Peng et al. [16] came up with a real-time detection method for tight butt joints based on laser structured light sensing and surface fitting. The double-line structured light was projected onto the welding seams, the optical trigonometric method was applied to obtain the 3D point cloud data of the welding seams, the moving least square method was used to iteratively fit the surface, and the pose of the welding seams were then calculated based on the relevant two-dimensional (2D) and 3D information. This method was used to consistently and accurately detect tight butt joints with a gap of less than 0.2 mm Yan et al. [17] proposed a method for automatically generating welding seam trajectories based on laser structured light scanning. The point cloud data of welded joints on a welded path was obtained by performing laser scanning on the workpieces, and the welding seam model of lap joints was accurately reconstructed using the cubic smooth spline algorithm. This method is especially applicable to complex curve welding seams with a gap of 0.2 mm, and shows great results with regard to path accuracy and the appearance of welding seams. 

As for the data for 3D welding seams, several effective extraction algorithms have been proposed. Patil et al. [18] suggested a 3D point cloud welding seam clustering extraction algorithm independent of the shapes of workpieces. With the point cloud data processing algorithm determined based on the point cloud library (PCL) point cloud database, Patil et al. extracted 3D welding seams, estimated the pose of the welding seams, and achieved the real-time seam tracking of butt joints using a five degrees of freedom (5-DOF) robotic arm. Based on information about the shapes of weldments, Yang et al. [19] proposed an offline 3D welding seam extraction algorithm based on point cloud segmentation. According to the spatial structure of the welding seams, a mathematical model was established to achieve offline path planning and pose estimation for 3D welding seams. However, 3D zigzag-line welding seams real-time tracking is still a challenge especially in high-speed welding. To overcome this problem, we proposed a method for the online extraction of the pose information based on laser displacement sensing and density-based clustering point cloud segmentation. In this paper, we introduce a method for the fast acquisition of the point cloud data for 3D zigzag-line welding seams (Section 2). Then, we discuss the point cloud segmentation (Section 3.1), extraction (Section 3.2), trajectory fitting (Section 3.3) and attitude estimation (Section 3.4). Finally, a welding experiment was performed to verify these methods (as described in Section 4), and the verification revealed the reliability of the system.

## 2. Fast Acquisition of Point Cloud Data for 3D Zigzag-Line Welding Seams

**Figure 2:**
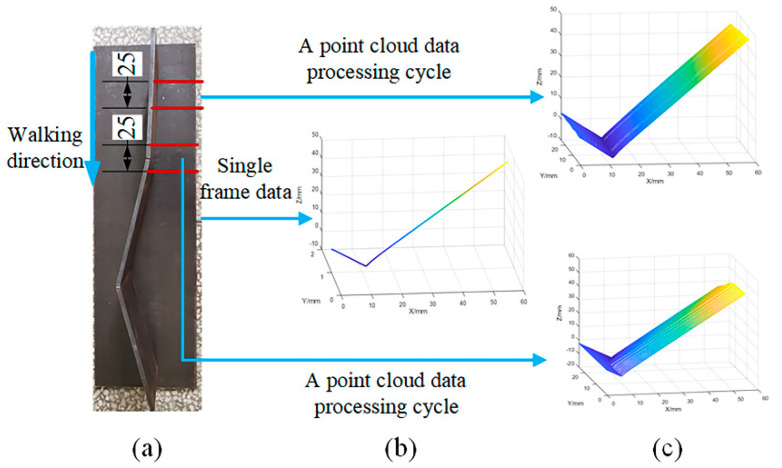
3D zigzag-line welding seam point cloud data; (**a**) Workpiece; (**b**) Single frame data; (**c**) Point cloud data

A laser displacement sensor was used to scan the workpiece (Figure 2a). The obtained data had 800 points for each frame, as shown in Figure 2b, with each value being the measured height of the measuring point of the workpiece. Each frame of data was stored in a 2D matrix. The column of the matrix corresponded to one frame of data measured at a particular time, with different columns corresponding to the measured data obtained at different times. The distance between adjacent columns (Δy) was determined by the welding velocity and time, and the distance between adjacent data for the same frame (Δx) was determined by the distance between the sensor and the workpiece, with the measuring height H being the coordinate value of $z_{c}$. The 2D matrix was converted into the 3D Cartesian coordinates $\left(x_{c},y_{c},z_{c}\right)$ by Equation (1). Figure 2c shows the point cloud data for the 3D zigzag-line welding seams.

(1) $$\{\begin{array}{c} x_{c}=r\Delta x\\ y_{c}=c\Delta y\\ z_{c}=H_{r,c} \end{array}$$

where r denotes the row and c represents the column of the point cloud data matrix.

## 3. Extraction of the Pose Information for 3D Zigzag-Line Welding Seams

**Figure 3:**
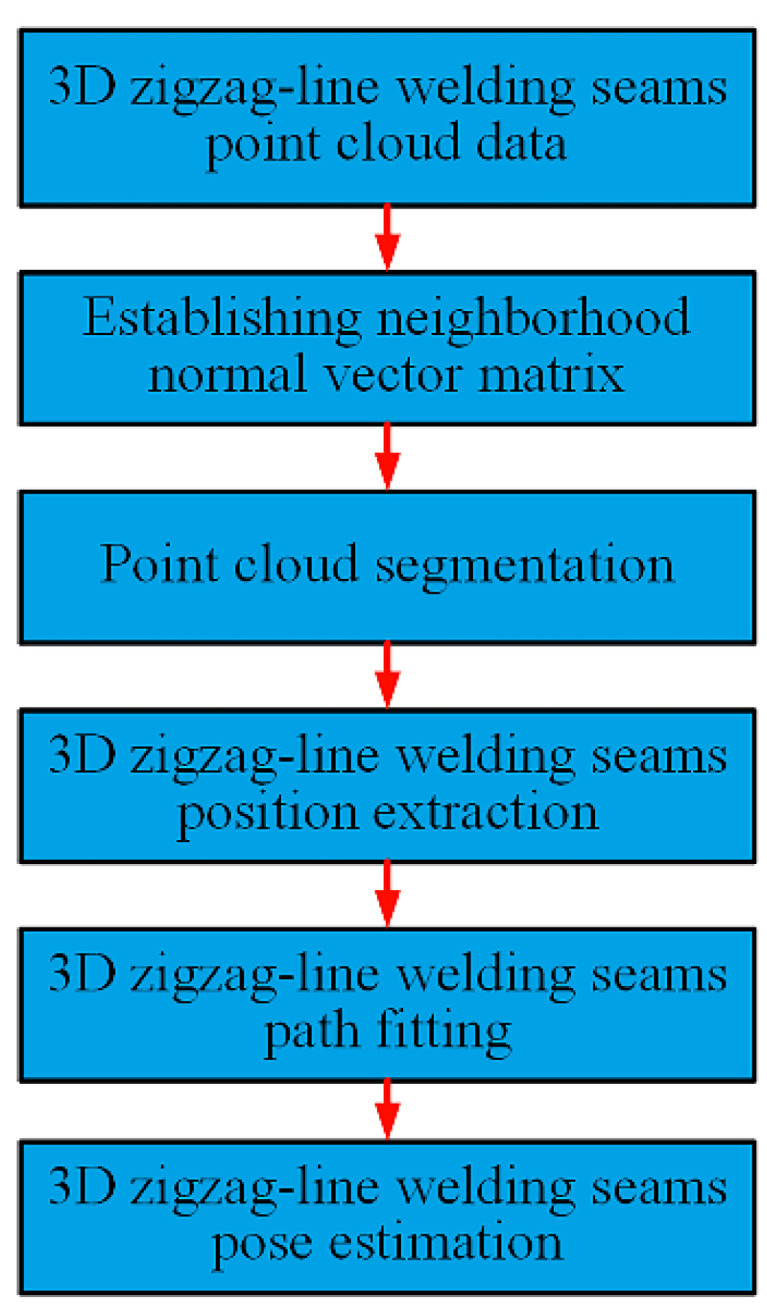
3D zigzag-line welding seams pose information online fast extraction process.

The 3D zigzag-line welding seams were assembled with a zigzag-line plate and a flat plate. To extract the pose information for the 3D zigzag-line welding seams, the position of the welding seams needed to be extracted in real-time, and the inflection point and the attitude of the welding seams needed to be estimated as well. The traditional point cloud feature extraction method could not be directly applied to extract the 3D zigzag-line welding seams. In this study, we proposed a novel method for the real-time extraction of pose information for 3D zigzag-line welding seams based on laser displacement sensor and density-based clustering point cloud segmentation. First, a method for establishing the neighborhood normal vector based on point cloud data was proposed. Second, the neighborhood normal matrix was segmented by the point cloud segmentation method based on the ρ-Approximate density-based spatial clustering of applications with noise (DBSCAN) algorithm [20] to gain the point cloud data for the plane and welding seams of the workpiece as well as the number of planes constituting the workpiece. Third, the random sample consensus (RANSAC) algorithm [21] was applied to extract the point cloud data for the 3D zigzag-line welding seams through the straight-line model and to perform trajectory fitting and attitude estimation, thereby extracting the pose information for the welding seams in real-time. Figure 3 shows the flow of the real-time extraction algorithm for the pose information for welding seams.

### 3.1. Point Cloud Segmentation

**Figure 4:**
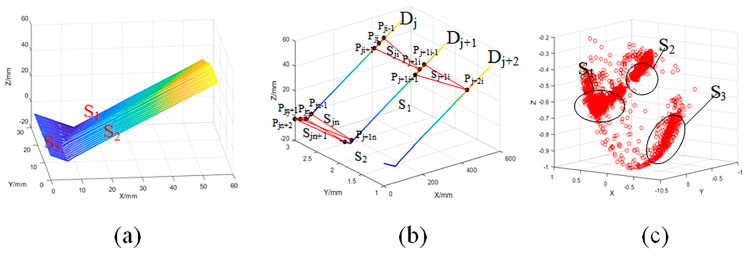
3D zigzag-line welding seams point cloud data neighborhood normal vectors; (**a**) Point cloud data; (**b**) Method for establishing neighborhood normal vectors; (**c**) Neighborhood normal vectors.

A method for establishing the neighborhood normal vector was proposed based on the characteristics of point cloud data for workpieces with 3D zigzag-line welding seams. As shown in Figure 4, the neighborhood plane was established based on Equation (2):

(2) $$S_{ji}=S_{p_{j,i-1},p_{j,i+1},p_{j+1,i}}$$

where $D_{j}$ denotes a frame for a data set sampled at the j-th time, $P_{j,i-1}$, $P_{j,i}$ and $P_{j,i+1}$ are three adjacent points in data set $D_{j}$, $P_{j+1,i}$ is the nearest point to $P_{j,i}$ on $P_{j+1,i}$, and $D_{j}$ and $D_{j+1}$ are two frames of adjacent data sets.

The normal vector for each data point was established based on Equation (3).

(3) $$Cov={\left[\begin{array}{c} p_{j,i-1}-p\\ p_{j,i+1}-p\\ p_{j+1,i}-p \end{array}\right]}^{T}\left[\begin{array}{c} p_{j,i-1}-p\\ p_{j,i+1}-p\\ p_{j+1,i}-p \end{array}\right]$$

where Cov is the covariance matrix corresponding to $P_{j,i}$, P refers to the geometric center of the point sets $P_{j,i-1}$, $P_{j,i}$, and $P_{j,i+1}$, and the eigenvector corresponding to the minimum eigenvalue of Cov is the neighborhood normal vector of $P_{j,i}$. Figure 4c shows the point cloud normal vectors of the 3D zigzag-line welding seams.

As shown in Figure 4c, the point cloud normal vectors are densely distributed in the same plane, while those in different planes are separated by a certain distance, of which the sparsely distributed points are the normal vectors of the points on the welding seams. Therefore, the point cloud segmentation of the 3D zigzag-line welding seams was achieved by clustering the neighborhood normal vectors using the density-based clustering method. DBSCAN [22] is a density-based algorithm typically used for the analysis of data structures, and it is able to obtain clusters for arbitrary shapes without having to specify the number of categories. Although the original DBSCAN algorithm features an ideal clustering effect, the running time cost is relatively high, especially when dealing with large-scale data, and the time complexity reaches O(n^2^) [23]. In this study, the ρ-Approximate DBSCAN algorithm was used for clustering analysis. As an approximate DBSCAN algorithm, the ρ-Approximate DBSCAN algorithm controlled the accuracy of the clustering results with the value of parameterρ, and it accelerated the clustering by dividing the data space into grids [20]. The efficient ρ-Approximate DBSCAN algorithm had a time complexity of O(n) under 3D point cloud conditions. The algorithm divided the data set into cclusters and sets of noise points; among the point cloud normal vectors of the 3D zigzag-line welding seams, the high-density area was distributed with plane point cloud normal vectors, and the point cloud normal vectors were distributed in the low-density area. Hence, the cclusters were the cplane normal data sets of the workpiece with 3D zigzag-line welding seams.

**Figure 5:**
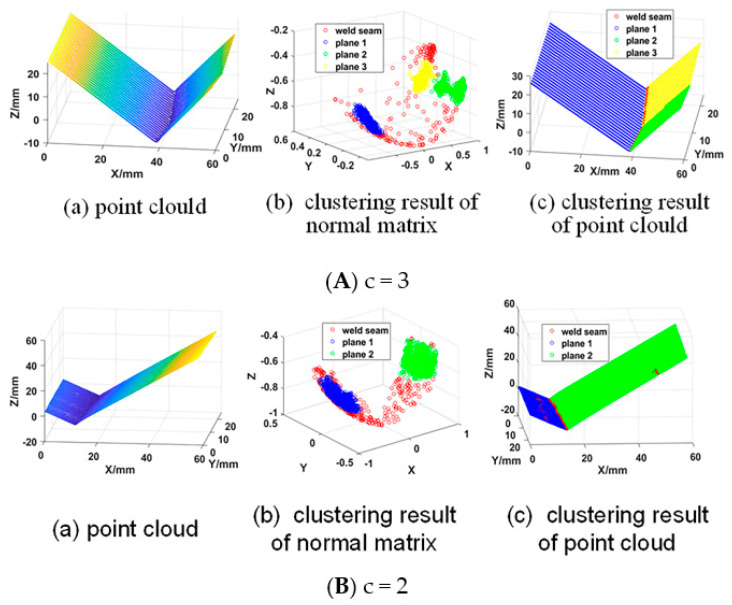
Point cloud segmentation results.

Figure 5 shows the segmented point cloud data. If there was an inflection point in the 3D zigzag-line welding seams, c = 3; if not, c = 2.

### 3.2. Extraction of Point Cloud Data of 3D Zigzag-Line Welding Seams

As shown in Figure 5, there were many noise points in the welding seam data set after point cloud segmentation. A method based on the RANSAC algorithm was used to extract the point cloud data for 3D zigzag-line welding seams from the noisy point cloud data in this study. Proposed by Fischler et al. [21], the RANSAC algorithm is a robust method based on testing. The RANSAC algorithm, which has powerful functions but a simple structure, is a method for estimating model parameters. The calculation models are shown in Equations (4) and (5).

(4) $$d_{i}=\frac{\left||(Data\left(i\right)-Data\left(1\right)\right)\times \vec{v}||}{\left|\right|\vec{v}\left|\right|}$$

(5) $$\left(x_{i},y_{i},z_{i}\right)=\{\begin{array}{c} \begin{array}{cc} inline\; & d_{i}\leq d_{thd} \end{array}\\ \begin{array}{cc} outline & d_{i}>d_{thd} \end{array} \end{array}$$

The welding seam data after point cloud segmentation served as the input data, where: $\vec{v}$ is the direction vector of the 3D line to be fitted, Data(i) refers to the i-th point cloud data, $d_{i}$ denotes the distance from any point of the point cloud to the fitted line, and $d_{thd}$ is the threshold for controlling the outliers in the point cloud. During the fitting of the point cloud data for the welding seams, the initial number of points was set as two, with the two points being Data(1) and Data(2), which could be used to determine the initial direction vector $\vec{v}=Data\left(2\right)-Data\left(1\right)$. The threshold $d_{thd}\;$ was set as 0.01 mm to control the outliers of the fitted line.

The process for the extraction of the point cloud of the 3D zigzag-line welding seams was as follows:

1. The point cloud was preprocessed using the bilateral filtering algorithm.
2. If c = 3, Steps 3, 4 and 5 would be implemented; if c = 2, only Step 3 would be implemented.
3. The RANSAC algorithm was used to extract the point cloud to obtain the point set $D_{1}$. Moreover, all the points belonging to $D_{1}$ were subtracted, and the remaining points were outlier.
4. The bilateral filtering algorithm was applied to process the outlier.
5. The RANSAC algorithm was used to fit the straight lines connected by the points in the outlier to obtain the point set $D_{2}$.

**Figure 6:**
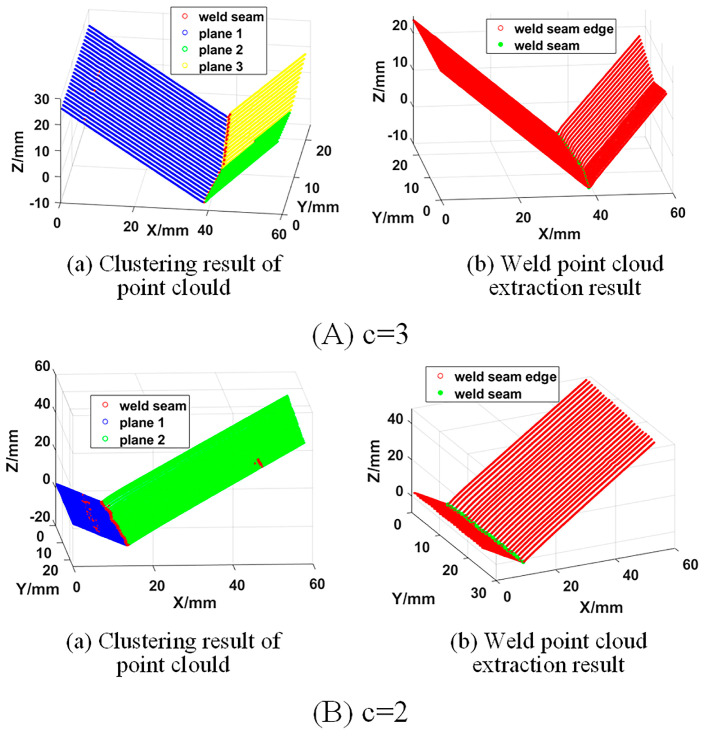
Weld point cloud extraction results.

As shown in Figure 6, the 3D zigzag-line welding seams were effectively extracted following the above steps.

### 3.3. 3D Zigzag-Line Welding Seams Trajectory Fitting

**Figure 7:**
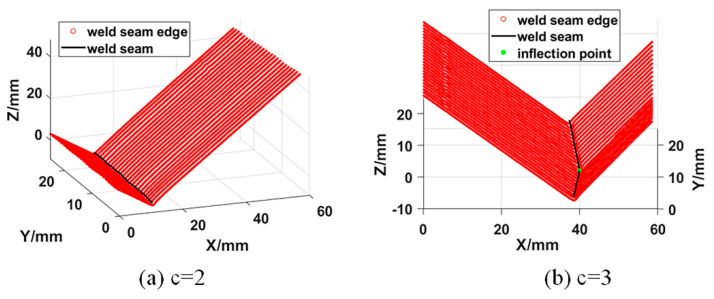
Welding seam trajectory fitting results; (**a**) When *c* = 2, the welding seam trajectory fitting result; (**b**) When *c* = 3, the welding seam trajectory fitting result.

The online extraction method for the 3D zigzag-line welding seams point cloud based on the RANSAC algorithm effectively realizes the task of welding seam extraction. The welding seam point cloud expresses the shape of the welding seam, but the welding seam trajectory is not smooth. In order to ensure the welding quality in the robot welding process, it is necessary to ensure that the welding robot welds smoothly along the welding seam trajectory to avoid severe vibration and shock. Therefore, the information extracted by the welding seam cannot directly guide the robot in the welding task, and planning the path of the welding robot is required to maintain the continuity of the displacement, speed and acceleration of the welding robot. Commonly used trajectory fitting methods include polynomial fitting and spline function fitting [24] In this paper, the moving least square method [25] was used to fit the 3D zigzag-line welding seams trajectory. The moving least squares fitting function f(x) is shown in Equation (6).

(6) $$f\left(x\right)=\overset{m}{\underset{i=1}{\sum }}\alpha_{i}\left(x\right)p_{i}\left(x\right)=p^{T}\alpha \left(x\right)$$

where $\alpha \left(x\right)={\left[\alpha_{1}\left(x\right),\alpha_{2}\left(x\right),\ldots ,\alpha_{m}\left(x\right)\right]}^{T}$ is the undetermined coefficient, which is a function of coordinate x.$\;p\left(x\right)={\left[p_{1}\left(x\right),p_{2}\left(x\right),\ldots ,p_{m}\left(x\right)\right]}^{T}$ is called the basis function, which is a complete polynomial of order k, and m is the number of terms of the basis function. Figure 7 shows the line segment and polyline segment weld trajectory fitting results of 3D zigzag-line welding seams. It can be seen that the fitting result of the weld trajectory is consistent with the shape of the actual weld trajectory.

### 3.4. Attitude Estimation of 3D Zigzag-Line Welding Seams

**Figure 8:**
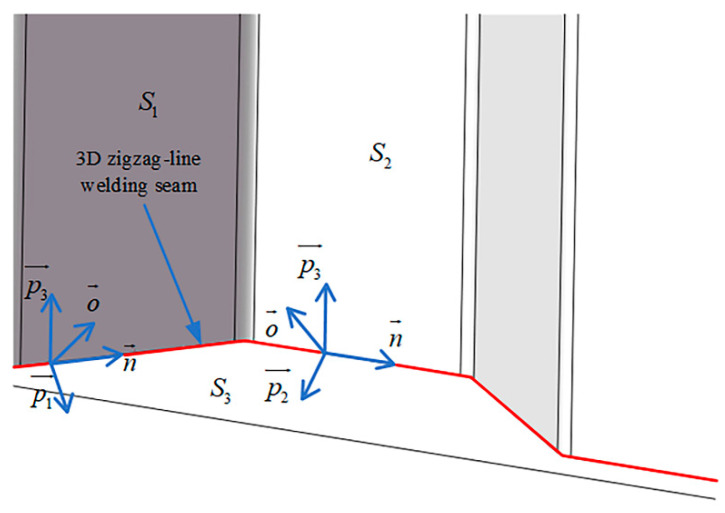
3D zigzag-line welding seam attitude model.

The 3D zigzag-line welding seam is composed of different polyline segments, and different polyline segments have different weld attitudes. In the actual welding process, due to the influence of complex welding conditions such as welding workpiece clamping, assembly and deformation, the attitude of the welding seam will change unpredictably. The attitude of the welding torch is also a key factor affecting the formation of the weld. The 3D zigzag-line welding seam attitude is shown in Figure 8, including the direction vector and normal vector. The calculation steps are:

1. If c=2, calculate $\vec{n}$, $\vec{o_{1}}$ by Formulas (7) and (8), where (x,y,z) is the point on the welding seam $L_{1}$.
2. If c=3, calculate $\vec{n}$, $\vec{o_{1}}$, $\vec{o_{2}}$ by Formulas (7)–(9), where (x, y, z) are points on $L_{1}$ and $L_{2}$ respectively.

(7) $$\vec{n}=\frac{\frac{df_{x}}{\partial t}i+\frac{df_{y}}{\partial t}j+\frac{df_{z}}{\partial t}k}{\left||\frac{df_{x}}{\partial t}i+\frac{df_{y}}{\partial t}j+\frac{df_{z}}{\partial t}k|\right|}$$

(8) $$\vec{o_{1}}=\frac{\vec{p_{1}}+\vec{p_{3}}}{\left||\vec{p_{1}}+\vec{p_{3}}|\right|}$$

(9) $$\vec{o_{2}}=\frac{\vec{p_{2}}+\vec{p_{3}}}{\left||\vec{p_{2}}+\vec{p_{3}}|\right|}$$

where $\vec{p_{1}}$, $\vec{p_{2}}$, $\vec{p_{3}}$ are the normal vectors of the weld points on the planes $S_{1}$, $S_{2}$, and $S_{3}$ respectively.

**Figure 9:**
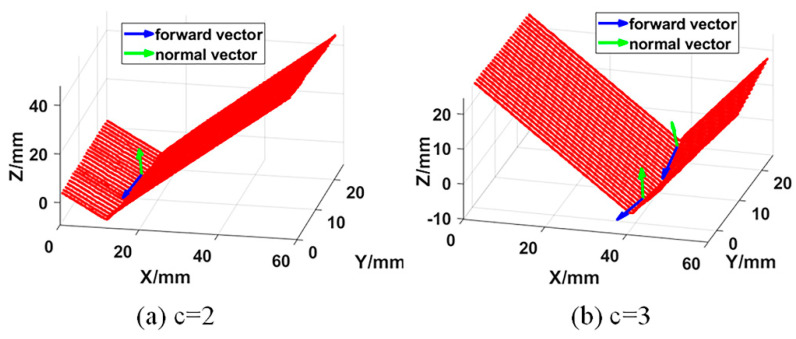
Welding seam attitude estimation results; (**a**) When c = 2, the welding seam attitude estimation result; (**b**) When c = 3, the welding seam attitude estimation result.

Figure 9 shows that 3D zigzag-line welding seam attitude online estimation can be realized. For a 3D zigzag-line welding seam workpiece, the welding robot can adjust the torch attitude online according to the welding seam attitude to ensure the welding quality.

## 4. Results and Analysis

### 4.1. System Platform

**Figure 10:**
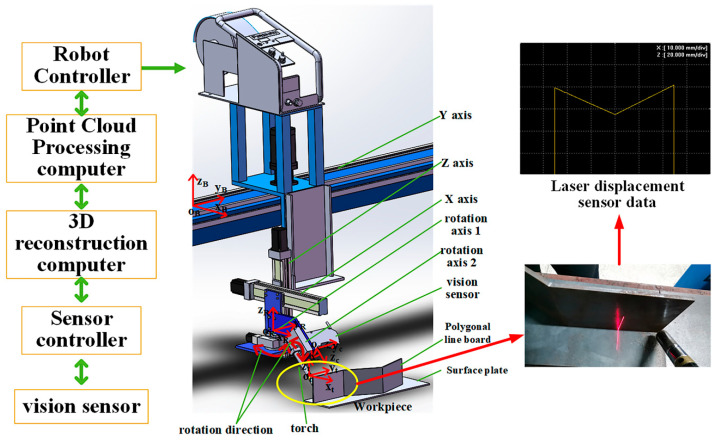
The structure of the experimental system.

As shown in Figure 10, the platform used in this study consisted of the welding robot system, the 3D reconstruction computer (Intel(R) Core (TM)i5–7300HQ CPU @2.5 G Hz, 8 G (RAM), (Lenovo, Beijing, China)), the point cloud data processing computer, and the laser displacement sensor (model LJ-G200 with controller model LJ-G5001(KEYENCE, Osaka, Japan)). The robot possessed five degrees of freedom (DOFs). During the welding process, the position of the welding torch was adjusted in the directions of the X, Y, and Z axes, the welding attitude was adjusted by rotating shafts 1 and 2, and rotating shaft 2 controlled the swinging of the welding torch to adapt to the swing welding process. Rotating shaft 2 and the laser displacement sensor were assembled on rotating shaft 1, and swing welding exerted no influence on the laser displacement sensor’s measurement of the workpiece. The laser displacement sensor was installed 50 mm ahead of the welding torch, the controller of the laser displacement sensor was connected to the 3D reconstruction computer that was connected to the point cloud data processing computer, and the point cloud data processing computer was connected to the robot controller. The laser displacement sensor obtained data for the welding seams through the real-time detection of the welding workpiece. The controller of the laser displacement sensor transmitted the data to the 3D reconstruction computer to three-dimensionally reconstruct the welding seams, and the 3D point cloud data was then transmitted to the point cloud data processing computer for further processing. The 3D pose information for the welding seams was transmitted to the controller of the welding robot so that the robot could complete the welding task.

To ensure the accuracy of the measured point cloud data, the method proposed by Zheng et al. [26] was used to calibrate the coordinate transformation relationship between the laser displacement sensor and rotating shaft 1. Equation (10) shows the transformation of the coordinate system of the sensor and the base coordinate system of the robot.

(10) $$\left[\begin{array}{c} x_{B}\\ y_{B}\\ \begin{array}{c} z_{B}\\ 1 \end{array} \end{array}\right]=T_{BR}T_{Rc}\left[\begin{array}{c} x_{c}\\ y_{c}\\ \begin{array}{c} z_{c}\\ 1 \end{array} \end{array}\right]$$

where $O_{B}X_{B}Y_{B}Z_{B}$ denotes the base coordinate system of the robot and $O_{c}X_{c}Y_{c}Z_{c}$ represents the coordinate system of the sensor. $T_{BR}$, as the transformation matrix between $O_{R}X_{R}Y_{R}Z_{R}\;$and $O_{B}X_{B}Y_{B}Z_{B}$, was obtained by the robot controller, and $T_{Rc}$, as the transformation matrix between $O_{c}X_{c}Y_{c}Z_{c}$ and $O_{R}X_{R}Y_{R}Z_{R}$, was obtained by calibration.

### 4.2. Experimental Verification

**Figure 11:**
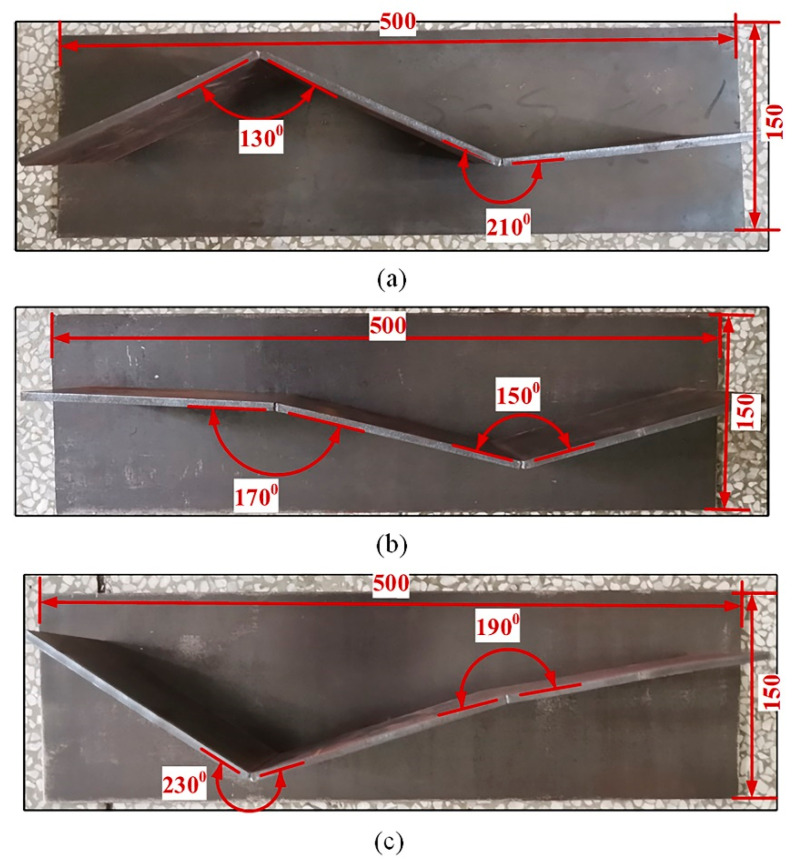
3D zigzag-line welding seam workpieces; (**a**) 3D zigzag-line welding seams with fold angles of 130°and 210°; (**b**) 3D zigzag-line welding seams with fold angles of 170°and 150°; (**c**) 3D zigzag-line welding seams with fold angles of 230°and 190°.

**Figure 12:**
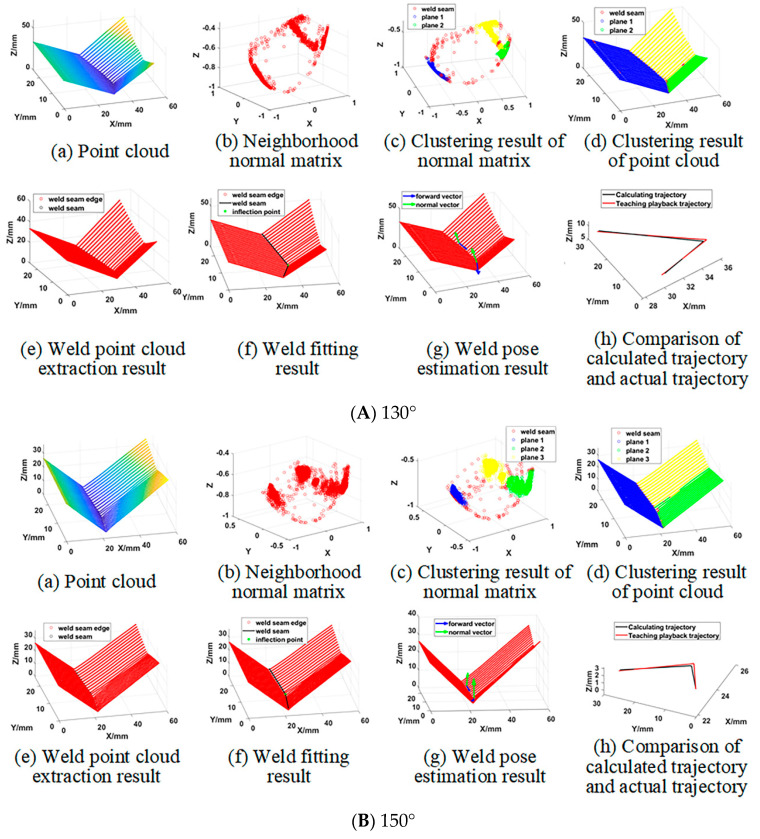

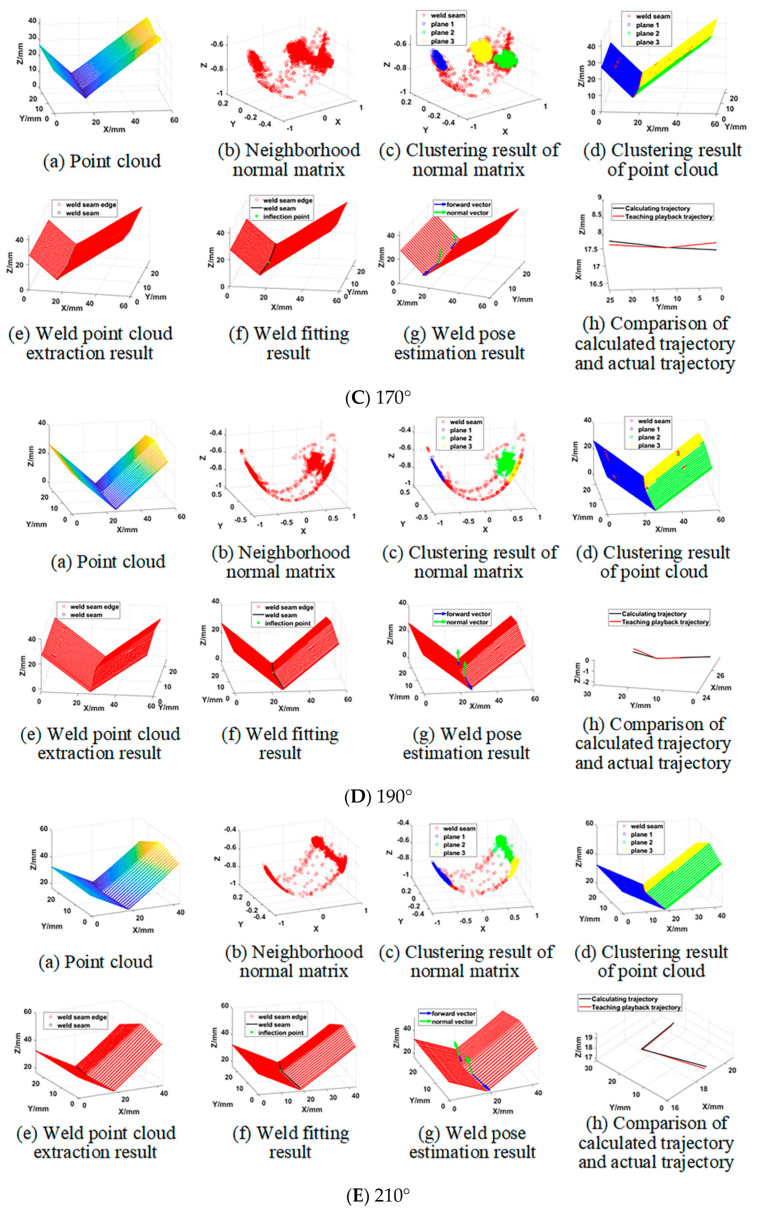

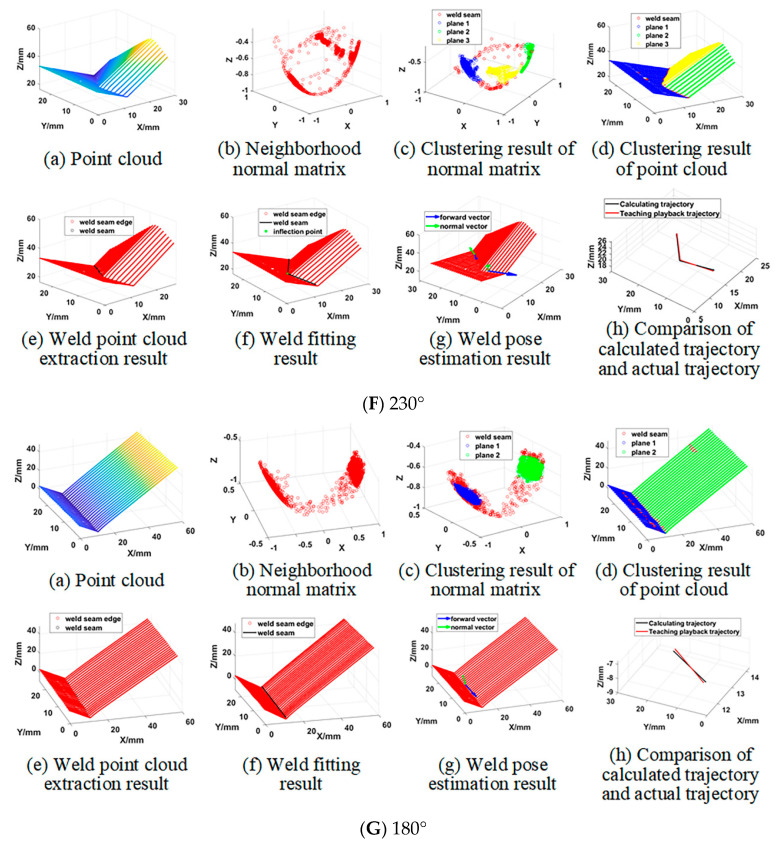
3D zigzag-line welding seam pose information online extraction results.

**Figure 13:**
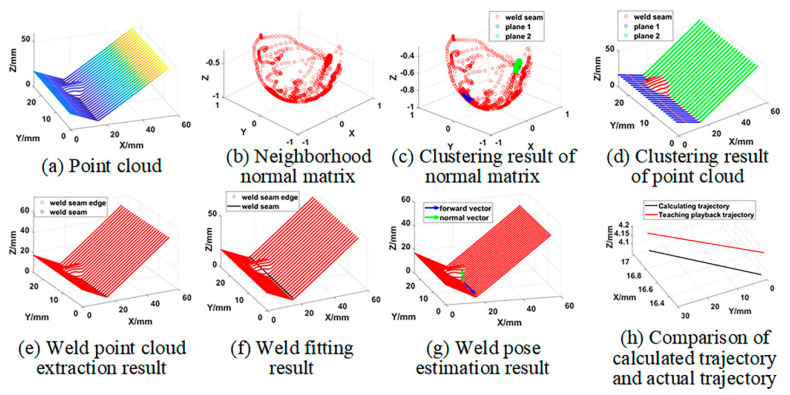
The influence of positioning welding seams on 3D zigzag-line welding seam pose information extraction.

**Figure 14:**
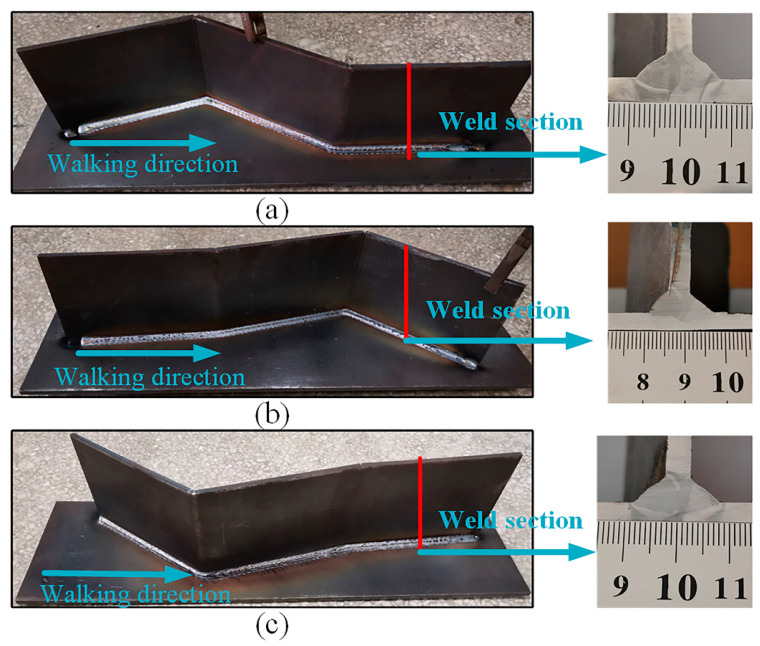
Real-time tracking results of 3D zigzag-line welding seams; (**a**–**c**) The real-time tracking results of 3D zigzag-line welding seams with fold angles of 130°, 150°, 170°, 180°, 190°, 210° and 230°.

To verify the effectiveness of the proposed method of quickly extracting the pose information for 3D zigzag-line welding seams for real-time welding seam tracking, welding experiments were carried out on 3D zigzag-line welding seams with fold angles of 130°, 150°, 170°, 180°, 190°, 210° and 230°. Figure 11 shows the workpieces with 3D zigzag-line welding seams. Table 1 shows the parameters of the welding process. In order to verify the accuracy of the on-line extraction of information about the 3D zigzag-line welding seams, the trajectories of the 3D zigzag-line welding seams were determined through accurate teaching before the experiment. In the welding process, the workpiece was measured by a laser displacement sensor, and the pose information of the 3D zigzag-line welding seams was extracted for the real-time tracking of 3D zigzag-line welding seams. Swing welding was used to improve the welding process, with the swing amplitude being 3 mm and the swing frequency being 3 Hz. Figure 12 shows the results processed by the algorithm with different fold angles. The 3D zigzag-line welding seams were assembled by the positioning welding seams before welding. To verify the influence of the positioning welding seams on the extraction of the pose information of 3D zigzag-line welding seams, a welding experiment was carried out on the 3D zigzag-line welding seams of typical positioning welding seams with a weld width of 10 mm and a welding length of 10 mm. The results of the experiment are shown in Figure 13. Figure 14 shows the effect of real-time tracking of the welding seams.

### 4.3. Error Analysis

We obtained the extraction error by comparing the data for the 3D zigzag-line welding seams read by the robot through accurate teaching with the points corresponding to the calculated values. With the maximum error (ME) and the mean square error (MSE) on the welding seams within a point cloud data processing cycle taken as the extraction errors, the calculation was conducted based on Equations (11) and (12). Table 2 shows the calculated errors. As shown in Table 2, the position error was less than 0.35 mm, the attitude error was less than 2 degrees, and the inflection point error was smaller than 0.5 mm.

(11) $$\{\begin{array}{c} X_{MSE}=\sqrt{\frac{\sum_{i=1}^{n}{\left(X_{c,i}-X_{t,i}\right)}^{2}}{n}}\\ Y_{MSE}=\sqrt{\frac{\sum_{i=1}^{n}{\left(Y_{c,i}-Y_{t,i}\right)}^{2}}{n}}\\ Z_{MSE}=\sqrt{\frac{\sum_{i=1}^{n}{\left(Z_{c,i}-Z_{t,i}\right)}^{2}}{n}} \end{array}$$

(12) $$\{\begin{array}{c} X_{ME}=\underset{i\in \left[1,n\right]}{\max}\left|X_{c,i}-X_{t,i}\right|\\ Y_{ME}=\underset{i\in \left[1,n\right]}{\max}\left|Y_{c,i}-Y_{t,i}\right|\\ Z_{ME}=\underset{i\in \left[1,n\right]}{\max}\left|Z_{c,i}-Z_{t,i}\right| \end{array}$$

where n is the number of points, $\left(X_{c,i},\;Y_{c,i},\;Z_{c,i}\right)\;$ are the calculated coordinates of the i-th point, and $\left(X_{t,i},\;Y_{t,i},\;Z_{t,i}\right)\;$ are the coordinates of the exact teaching of the i-th point.

### 4.4. Efficiency of the Algorithm

To test the effectiveness of the algorithm, the efficiency of the algorithm for the online extraction of 3D zigzag-line welding seams was tested on a computer with MATLAB 2019 (MathWorks, Natick, MA, USA) as the testing platform. Two computers with Intel (R) Core (TM) i5-7300HQ CPU @2.5 GHz, 8 G (RAM) as the configuration served as the 3D reconstruction computer (TDRC) and the point cloud data processing computer (PCDPC). Two computers worked at the same time. The TDRC performed a three-dimensional reconstruction on each frame of data obtained by the laser displacement sensor at a particular time interval ($t_{interval}=\frac{1\mathrm{mm}}{v_{w}}$), and transmitted the data to the PCDPC after completing the three-dimensional reconstruction of 25 frames of data. The PCDPC completed the extraction of weld pose information and waited for the next data transmission from the TDRC. The maximum time the main algorithm takes for multiple runs is shown in Table 3. The processing and extraction time of the welding seams, t had to satisfy t≤T. $T=\frac{0.5d_{f}}{v_{w}}$, where $v_{w}$ is the welding velocity, $d_{f}=50\;\mathrm{mm}$ and $v_{w}=1500\;\mathrm{mm}/\min$. To increase the data processing speed and reduce the data density, one frame of data was collected at intervals of 1 mm. The data was collected at a particular interval ($t_{interval}=\frac{1\mathrm{mm}}{v_{w}}$), and $t_{rcn}$, the 3D reconstruction time of each frame of data, had to meet the condition $t_{rcn}<t_{interval}$. As shown in Table 3, the extraction time was less than 120 ms, which satisfied t≤T. It took 35 ms for TDRC to process a frame of data, which was less than the sampling interval (40 ms). It was thus clear that the proposed method met the requirements for the online extraction of 3D zigzag-line welding seams in terms of the running time.

## 5. Discussion

The online extraction system of pose information of 3D zigzag-line welding seams proposed in this study was used to carry out welding experiments on 3D zigzag-line welding seams for typical fold angles. The online extraction of the pose information was achieved for 3D zigzag-line welding seams with a fold angle ranging from 130° to 230°. 3D zigzag-line welding seams with a fold angle that was too large or too small interfered with the laser displacement sensor in acquiring the point cloud. In the future, we will design a more adaptive laser displacement sensor installation mechanism to adapt to 3D zigzag-line welding seams with a larger range of fold angles.

A verification experiment was carried out to extract the pose information for 3D zigzag-line welding seams with positioning welding seams, the results of which are shown in Figure 13. The point cloud data for the positioning welding seams was filtered as noise. For typical positioning welding seams with a weld width of 10 mm and a welding length of 10 mm, the accuracy of the online extraction of pose information of the 3D zigzag-line welding seams was not affected. Nevertheless, without detecting the size characteristics of the positioning welding seams, the proposed method failed to provide any guidance on the adaptive welding process. In a future study, we will add the function of identifying the size of positioning welding seams of 3D zigzag-line welding seams in order to provide a basis for adaptive welding.

In the experiment, the 5-axis robot was used to realize the online fast extraction of 3D zigzag-line welding seam pose information, and the method has good generalization. This method can be applied to different scenarios by using the point cloud processing computer to realize the communication with different robot controllers, such as the welding of large structure workpieces. In the future, we will do further research work in this area.

## 6. Conclusions

In this study, we proposed a method for the online extraction of the pose information for 3D zigzag-line welding seams for the real-time tracking of welding seams, and we made the following conclusions:

(1) An online extraction system for the pose information of 3D zigzag-line welding seams was successfully established for the real-time tracking of welding seams
(2) A 3D zigzag-line welding seam point cloud segmentation method based on the ρ-Approximate DBSCAN clustering algorithm was used to achieve the online segmentation of the point cloud data of 3D zigzag-line welding seams. The running time of the main algorithm is less than 120 ms, which meets the requirement for the online extraction of welding seam pose information for high-speed welding with a welding speed exceeding 1500 mm/min.
(3) A number of welding experiments were carried out on 3D zigzag-line welding seams with a fold angle ranging from 130° to 230°. The results of the experiments showed that when the welding velocity was 1000 mm/min, the proposed method achieved a welding seam position detection error of less than 0.35 mm, and a welding seam attitude estimation error of less than 2 degrees. This met the requirements for the online extraction of the pose information for 3D zigzag-line welding seams for the real-time tracking of welding seams.
(4) The proposed method was applicable to swing welding. The method is expected to be extensively used in the welding of middle thickness plates during the manufacturing of marine engineering equipment, heavy lifting equipment, and logistics transportation equipment.

## Figures and Tables

**Table 1:** Welding process parameters.

Parameters	Value
Welding method	gas metal arc welding (GMAW)
Welding voltage (V)	28
Welding current (A)	290
Welding speed (mm/min)	1000
Wire diameter (mm)	1.2
Wire extension (mm)	12
Welding materal	Q235
Thickness of the workpiece (mm)	5
Shielding gas	80%Ar + 20%CO_2_

**Table 2:** Welding seam extraction error.

Weld Seam	Error	X (mm)	Y (mm)	Z (mm)	Forward Vector (Degree)	Normal Vector (Degree)
Straight line	maximum error (ME)	0.32	0.24	0.3	1.8	1.9
Straight line	mean square error (MSE)	0.15	0.13	0.19	1.4	1.6
Polygonal line	ME	0.31	0.33	0.22	1.9	1.6
Polygonal line	MSE	0.14	0.18	0.13	1.2	1.1
Inflection point	error (E)	0.41	0.46	0.42		

**Table 3:** The algorithm running time.

Key Steps	Running Time (ms)
3D reconstruction	35 (3D reconstruction computer, One frame of data processing time)
Point cloud segmentation	80 (point cloud data processing computer (PCDPC))
Feature extraction	25 (PCDPC)
Path fitting	15 (PCDPC)
The total time	120 (PCDPC)

## Data Availability

Data sharing not applicable.
